# Prevalence of infratentorial superficial siderosis in a large general population sample from the UK Biobank

**DOI:** 10.1007/s00415-025-12965-w

**Published:** 2025-03-04

**Authors:** N. Kharytaniuk, D. Hikmat, H. Ozkan, E. A. Lim, D. E. Bamiou, P. Cowley, H. R. Jäger, D. J. Werring

**Affiliations:** 1 UCL Ear Institute, University College London, 332-336 Gray’s Inn Road, London, WC1X 8EE UK; 2 National Institute for Health and Care Research University College London Hospitals Biomedical Research Centre (Deafness and Hearing Problems Theme), London, UK; 3 Department of Neuro-Otology, Royal National ENT and Eastman Dental Hospitals, London, UK; 4 Stroke Research Centre, Department of Brain Repair and Rehabilitation, Queen Square Institute of Neurology, University College London, London, UK; 5 Department of Radiology, Royal Free Hospital, London, UK; 6 Lysholm Department of Neuroradiology, National Hospital for Neurology and Neurosurgery, Queen Square, London, UK; 7 Department of Imaging, Imperial College Healthcare NHS Trust, London, UK; 8 Neuroradiological Academic Unit, Queen Square Institute of Neurology, University College London, London, UK; 9 Comprehensive Stroke Service, National Hospital for Neurology and Neurosurgery, Queen Square, London, UK

**Keywords:** Superficial siderosis, Infratentorial, Prevalence, UK Biobank

## Abstract

**Background:**

Classical infratentorial superficial siderosis (iSS) is considered a rare but potentially disabling neurological disorder. It results from slow chronic blood extravasation into the cerebrospinal fluid and deposition of the iron degradation product hemosiderin within the subpial layers of the central nervous system. Susceptibility-weighted (SW) magnetic resonance imaging (MRI) is the reference diagnostic modality. Few studies have described the population prevalence of cerebellar or infratentorial siderosis, and there are none from the UK population. The aim of this cross-sectional observational study was to estimate the prevalence of iSS in the UK Biobank sample using pre-defined radiological criteria.

**Methods:**

We reviewed SW MRIs of participants from the UK Biobank, looking for the radiological features of classical iSS: involvement of infratentorial structures (superior vermis, cerebellar folia, brainstem, or craniocervical junction). We calculated the point prevalence as the number of identified cases per total number of cases reviewed and 95% confidence intervals (CI) using Wilson’s Score formula.

**Results:**

Of 10,305 SW MRIs reviewed, five cases with radiological features of iSS were identified demonstrating cerebellar/superior vermis involvement. The estimated prevalence of iSS was calculated as 48.5 (95%CI 20.7–113.5) cases per 100,000 population.

**Conclusions:**

This is the first study to estimate iSS prevalence in the UK population. The prevalence of iSS is higher than that reported for other rare neurological and neuro-otological disorders, suggesting an important unmet healthcare need for early diagnosis and targeted management strategies. Further studies are needed to determine the clinical associations and prognostic significance of radiologically defined iSS in the general population.

## Introduction

Since its first description in 1908, classical infratentorial superficial siderosis (iSS) of the central nervous system (CNS) has been reported in over 270 cases [1–3]. Other forms of superficial siderosis have been subsequently identified and the nomenclature for superficial siderosis was proposed and published elsewhere [4, 5]. In contrast to other forms of superficial siderosis, classical iSS (synonymous with Type 1 iSS) is characterized by the deposition of iron degradation products (predominantly in the form of hemosiderin) following chronic intermittent or continuous and low volume extravasation of red cells into the cerebrospinal fluid affecting infratentorial structures of the CNS with or without additional supratentorial involvement [3, 4]. These deposits can be visualized in vivo using magnetic resonance imaging (MRI) [6]. For the purposes of this study, we will refer to the classical iSS (Type 1) as “iSS”.

The advent of MRI, its increasing availability, and particularly the introduction of hemorrhage-sensitive sequences have contributed to the increased recognition of iSS on imaging and thus its increased incidence and reporting in recent decades [2, 3, 6]. Hemorrhage-sensitive sequences can better demonstrate signal changes in iron-laden tissues than T2-weighted sequence, due to blooming effect, and are therefore preferred for visualizing hemosiderin [4, 5, 7, 8].

Recently published iSS radiological diagnostic criteria describe the involvement of the cerebellum, brainstem (including medulla, pons, and midbrain) and craniocervical junction [4]. Involvement of superior cerebellar vermis is hypothesized to be an early radiological feature of iSS [5].

iSS is considered rare, but its prevalence is currently unknown [9]. It is listed in the OrphaNet Rare Diseases Registry under “Superficial siderosis of the CNS” (ORPHA:247,245) [9]. The definition of “rare disease” varies between regions [10]. The European Union defines rare as “affecting fewer than five individuals per 10,000 people in a general European population”, or one per 2000 population [10, 11]. The prevalence of iSS has been studied in a small number of hospital- and community-based studies from the United States, Austria, the Netherlands, and Germany. They reported the prevalence between 0.03% and 0.14% (30.7–141.6 individuals per 100,000 population) [12–15]. The wide variation in the prevalence range reported in these studies may be due to the difference in diagnostic criteria and imaging protocols used, study populations, and access to healthcare facilities across various geographical regions (Table 1). Most of these studies described “cerebellar” or “infratentorial” siderosis in their cohorts and were published before the proposed radiological diagnostic criteria [12, 14, 15].

**Table 1:** Study findings in the context of iSS prevalence reported in other studies

Referenced study	Year	Population (country)	MRI parameters	Findings	Percent prevalence (95% CI)	Prevalence per 100,000 population
Current study	2022	Community (UK)	3 T SWI	5 in 10,305	0.0485 (0.0207–0.1135)	48.5
Friedauer, et al. [13]	2020	Hospital (Germany)	T2-weighted; T2*GRE/SWI‡	30 in 97,733	0.0307 (0.0215–0.0438)	30.7
Offenbacher, et al. [15]	1996	Hospital (Austria)	1.5 T T2-weighted; T2*GRE	9 in 8843	0.1018 (0.0536–0.193)	101.8
Pichler, et al. [14]	2017	Community (USA)	3 T T2*GRE	2 in 1412	0.1416 (0.0389–0.515)	141.6
Vernooi,j et al. [12]	2009	Community (Netherlands)	1.5 T T2*GRE	1 in 1062	0.0942 (0.0166–0.5314)	94.2
Pooled findings				47 in 119,355	0.0394 (0.0296–0.0524)	39.4

*CI* confidence intervals, *GRE* gradient recalled echo, *MRI* magnetic resonance imaging, *SWI* susceptibility-weighted imaging, *3 T/1.5 T* 3/1.5 Tesla field strength

^‡^field strength not stated

The UK Biobank (UKB) is a large-scale prospectively collected health data repository from a population sample of over 500,000 participants recruited across the UK [16, 17]. The age range of the UKB participants was 40–69 years which is similar to the age at which iSS is typically diagnosed [3, 4, 17]. MRI acquisition for up to 50,000 participants including hemorrhage-sensitive (susceptibility-weighted, SW) sequences was undertaken between 2014 and 2022 [18–20]. We aimed to estimate the prevalence of iSS in the UKB sample using pre-defined radiological criteria [4, 16–19].

## Methods

### UKB data access and ethical and data permissions

The National Health Service (NHS) Research Ethics Service granted approval for the UKB study (Ref 11/NW/0382; 16/NW/0274; 21/NW/0157); all participants gave informed consent [17, 18]. The UKB Access Committee granted formal access to the UKB data under Project Application Number 16256. Standardized protocol for data acquisition was followed [20].

### Study design and setting

This was a single-rater cross-sectional observational study that used pre-collected unlabeled imaging data from a dedicated health-related and imaging data repository (UKB). Inter-rater reliability calculations were not performed.

#### UKB population

The imaging data had been collected from predominantly healthy and pre-symptomatic individuals aged 40–69 years who had been recruited from the general UK population for the UKB study between 2006 and 2010. Individuals who were registered with the National Health Service who were residents within ≤ 25 miles from the study assessment centers were invited to participate; invitations were sent out by post [21]. Demographic and health-related data were collected as part of the UKB data acquisition at baseline. An in-depth neurological evaluation was not performed. Of the 500,000 UKB participants, 50,000 participants underwent a single MR imaging session between 2014 and 2022 (and within 9.6 ± 1.1 years after the initial recruitment).

#### Inclusion/exclusion criteria

Included in this study were the data from the participants who underwent MRI Brain with SW sequences. We excluded the imaging data (from 35 participants) that were of insufficient quality to reliably ascertain if the signal dropout was present or absent on SW sequences, for example due to motion artifacts precluding the visualization of key infratentorial regions. Such images were deemed non-diagnostic for iSS. Data on demographics and health-related variables were not accessed as part of this study.

### UKB imaging acquisition and anonymization

UKB brain imaging was acquired at different sites using identical 3 Tesla field strength Siemens Skyra MRI scanners (software platform VD13) with standard 32-channel head coils [19, 20]. Hemorrhage-sensitive (SW) sequence images were acquired using a three-dimensional (3D) dual gradient-echo sequence with the following parameters: voxel size = 0.8 × 0.8 × 3 mm^3^, matrix size = 256 × 288 × 48 (whole-brain coverage), echo times (TE1/TE2) = 9.4/20 ms, repetition time (TE) = 27 ms, in-plane acceleration = 2 and total scan time = 2 min 34 s [19, 20].

Automated image processing pipelines were created to remove artifacts and for between-participants compatibility of images [20]. To ensure anonymization of the UKB data, brain images were defaced [20]. The images were available in DICOM (Digital Imaging and Communications in Medicine) and NIFTI (Neuroimaging Informatics Technology Initiative) formats.

### Accessing and viewing UKB imaging data

Consecutive UKB MR brain images were sequentially downloaded from the UKB server. At the time of the study, 15,000 MR brain images of 50,000 available were downloaded. Axial SW images, with default standard UKB windowing, were reviewed for the presence of hypointense regions consistent with the iSS pattern of hemosiderin distribution based on the pre-defined radiological diagnostic criteria [4]. The imaging files were accessed remotely and were viewed in semi-dark conditions on the LG UltraFine 4 K display monitor (2019, LG UK), in NIFTI format using pre-installed MRIcron imaging viewer software program (Neuroimaging Tools and Resources Collaboratory, University of South Carolina, v1.0.20190902, 2019) [22].

### Quality control measures

The first author (NK) had a two-year experience of reviewing clinical imaging of patients with known iSS at the specialist superficial siderosis multidisciplinary team meetings at our tertiary clinical center and underwent dedicated training by the specialist clinical neuroradiologists on the team (HRJ, PC and EAL) in reviewing the neuroimaging for the presence of signal changes suggestive of hemosiderin deposits.

Radiological findings suggestive of iSS were co-verified with at least two senior research team members, including least one senior neuroradiologist with experience in iSS, and using appropriate windowing. Corresponding T2-weighted images of the identified iSS cases were reviewed to rule out other causes for signal dropout and hemosiderin deposits.

### Statistical analysis

Sample size calculations were performed using the following formula [23]:

$$n=\frac{{\text{z}}^{2}\text{p}(1-\text{p})}{{d}^{2}}$$

where *n* = sample size; *z* = level of confidence; *p* = expected prevalence; *d* = margin of error (precision).

To determine the sample size needed to estimate iSS prevalence with 95% confidence level and 0.1% margin of error, based on the highest prevalence of infratentorial siderosis (0.1416%) previously reported by Pichler et al. [14], the minimum required sample was 5432, calculated using the Scalex SP Calculator [23, 24]. Calculating the sample size, needed to estimate the prevalence with 99% confidence level and 0.1% margin of error, the minimum required sample was 8578 [23, 24]. To calculate the prevalence of iSS within the studied sample, we used the following formula [25, 26]:

$$\text{Prevalence }\left(\text{cases}\right)\uprho =\frac{\text{number of identified cases in the sample}}{\text{total number of cases in the sample}} \times 100$$

The estimated 95% confidence intervals (CI) for the prevalence were calculated using Wilson’s Score method available from the Public Health England tool for calculating common public health statistics, and ‘OpenEpi Collection for Epidemiologic Calculations’ software (v3.01, 2013, Emory University, Atlanta, USA) [25–27].

## Results

Of the 50,000 MRI Brain scans available as part of the UKB imaging data at the time of this study, the first 10,340 MRI scans were downloaded and reviewed as part of this study. Thirty-five scans had severe motion artifact and were excluded from the analysis.

### Imaging features of identified cases

**Fig. 1:**
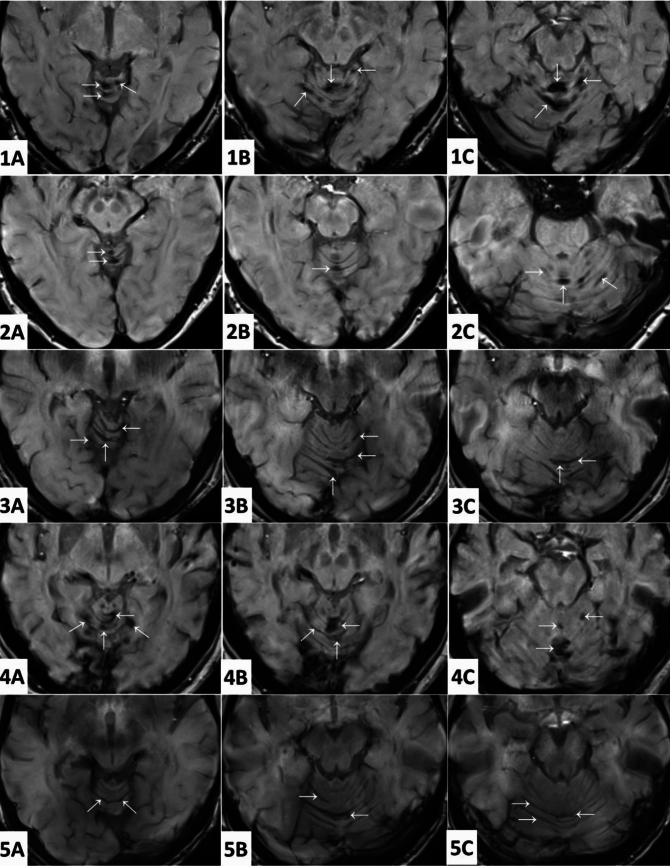
Axial susceptibility-weighted magnetic resonance images, with typical findings suggestive of iSS. (Cases 1–5). Cephalad to caudal appearances (**A**–**C**) of superior vermis and cerebellar folia. Hypointense signal changes suggestive of hemosiderin deposits are marked with arrows. Source: UK Biobank ©. Reproduced by kind permission of UK Biobank ©

**Fig. 2:**
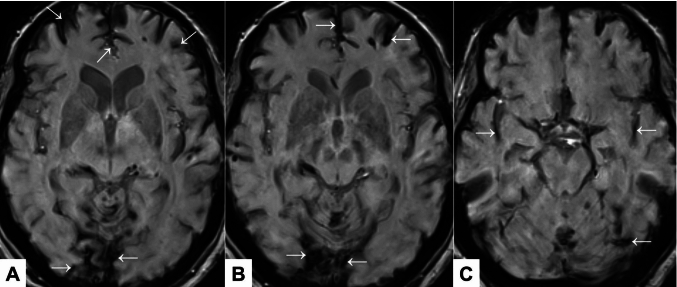
Axial susceptibility-weighted magnetic resonance images of supratentorial involvement (Case 4). Appearances of hypointense regions (cephalad to caudal, **A**–**C**) suggestive of hemosiderin deposits are marked with arrows. Source: UK Biobank ©. Reproduced by kind permission of UK Biobank ©

Of the 10,305 cases analyzed, five had radiological features suggestive of iSS with the involvement of the superior vermis and cerebellar folia (Fig. 1). The radiological appearances were similar to those observed in patients during clinical practice. In addition to the infratentorial involvement, hypointense areas consistent with hemosiderin deposits were observed in the supratentorial distribution symmetrically involving the cerebral convexities, in one case (Fig. 2).

### iSS prevalence in the studied sample

The iSS prevalence was calculated as the proportion of the five cases identified out of 10,305 cases reviewed ρ = 0.0004852 or 0.0485% (95% CI 0.0207 to 0.1135), or 48.5 (95% CI 20.7–113.5) individuals per 100,000 population (Table 1).

## Discussion

This is the first study to estimate iSS prevalence in a large cohort of individuals representing a cross-sectional sample of the general UK population aged 40–69, with the participants’ age-range being similar to the age-range at which iSS is commonly diagnosed [3].

To identify the cases with radiological features of iSS, we used the hemorrhage-sensitive (SW) MR imaging which is known to be more sensitive for the detection of signal changes consistent with hemosiderin-laden tissues, including microhemorrhages [4, 7, 8].

**Fig. 3:**
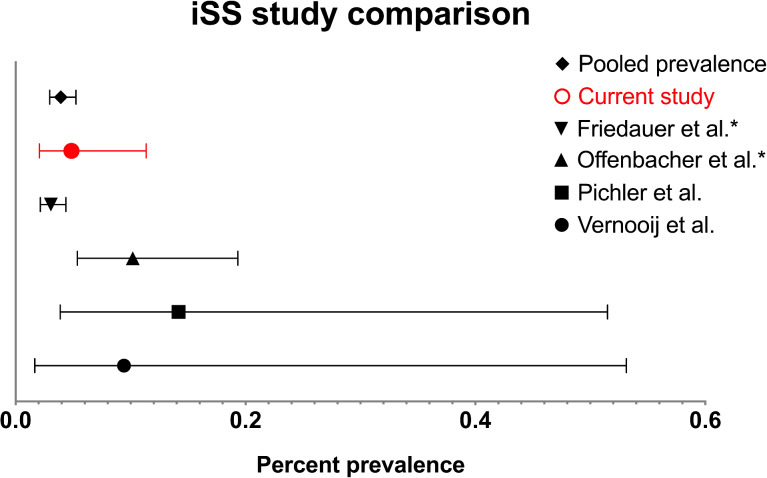
Between-study comparison of estimated iSS prevalence. The findings from current study and calculated pooled prevalence included, mean with error bars provided [12–15]. *Hospital-based studies

Our findings suggest that iSS might not be as rare as previously hypothesized, albeit still, at 48.5 per 100,000, falling just within the range of the definition of a rare disease in a European population [11], and appears similar to the prevalence of superficial siderosis reported in other studies to date (Table 1**, **Fig. 3) [12–15].

Our clinical experience of patients with iSS who have minimal symptoms led us to hypothesize that the radiological findings may precede the onset of clinical manifestations associated with iSS. The observed invariable involvement of the superior cerebellar vermis and folia, in keeping with the radiological diagnostic criteria [4], in a randomly selected and presumably asymptomatic population suggests that these regions are likely to be involved early in the disease course of iSS [5]. Although cerebral amyloid angiopathy (CAA) has been reported to cause cerebellar siderosis, we did not observe lobar cerebral microbleeds or asymmetrical cortical superficial siderosis, suggesting that this was not the cause of the iSS we observed.

Table 2 demonstrates the prevalence of other rare neurological and neuro-otological disorders. The prevalence of iSS estimated in our study appears higher than these conditions. This highlights the need of the clinicians to be aware of iSS as an entity that may be encountered in the clinical setting, at least as often as other rare but well-recognized neurological disorders (e.g., motor neuron disease). Our findings suggest an important potential unmet healthcare need, with potential implications for resource allocation, both in clinical and research settings including provision of funding for healthcare including diagnosis and management, and the development of novel therapeutic targets.

**Table 2:** Prevalence of the rare disorders likely to be encountered in the neurology, neuro-otology, audiovestibular medicine and ENT (ear, nose and throat) clinics

Neurological disorder	Prevalence per 100,000 population	Region/country (reference)
Classical (type 1) infratentorial superficial siderosis	39.4 48.5	Pooled finding from current and previous studies Current study
Amyotrophic lateral sclerosis (ALS)	4.4 – 6 1.1 – 9.5 1.0 – 11.3	USA [32, 33] Europe [34, 35] Asia/Pacific [34, 36]
Cardiac arrhythmias, neuropathy and vestibular areflexia syndrome (CANVAS)	5	European ancestry, UK [37]
Spinocerebellar ataxia, autosomal dominant types (SCA3, SCA2, SCA6, SCA7)	1–5 0.9–3	Global [38, 39] Europe [40]
Friedreich’s ataxia (FRDA)	0.3–5	Europe [41]
Vestibular schwannoma (VS)	12–42	USA [42], Minnesota, USA [43]

iSS can be encountered in a general neurology clinic, albeit infrequently, and should be suspected in individuals presenting with often a constellation of hearing loss, ataxia, imbalance, or symptoms of myelopathy, and likely with the history of previous CNS trauma or surgery or in some cases of spontaneous intracranial hypotension. Hemorrhage-sensitive sequences should be included in the MR imaging studies as part of the radiological work-up, and radiologists should be alerted to the possibility of iSS as anecdotally, it has been missed on MRI. iSS radiological diagnostic criteria and clinical care pathways have been proposed by our group and are available elsewhere [4, 5].

The strength of our study is that we have included the largest sample to date to estimate the iSS prevalence from a non-hospital population. This is also the first study to estimate the point prevalence of iSS in a UK population sample. We used standardized SW MR imaging and applied pre-defined diagnostic radiological criteria [4]. The single-rater study design ensured consistency, with necessary training and support provided by the expert neuroradiological team and adjudication of uncertain cases and agreement reached by consensus.

There are several limitations to this study. This is a single-rater cross-sectional study of the radiological findings consistent with iSS. Inclusion of participants’ demographics, self-reported symptoms, health-status, and the audiological data was not possible due to lack of data access, so we were unable to correlate these data with the radiological findings. The cross-sectional study design does not permit longitudinal data collection and cases, in which iSS may have developed subsequently, might have been missed. Nevertheless, our study provides useful data about prevalent iSS cases in the UK. We are aware of two longitudinal studies which reviewed iSS radiological baseline and interval findings to quantify changes in response to iron-chelating therapy [28, 29]. We are not aware of any studies investigating the incidence of new cases of iSS. Dedicated longitudinal prospective studies are needed that focus on the natural history of iSS and how frequently new cases develop, that track correlation between the radiological and clinical findings and subgroup analyses for potential risk factors or demographic patterns.

Our study relies on the single-rater review of SW MR images which may have introduced further design bias and thus influenced the findings. The corresponding T2-weighted MR images of the identified cases were reviewed and findings confirmed with senior neuroradiology team members. We recognize that parallel and blinded multi-rater analysis of both SW and T2-weighted MR images may have improved the sensitivity of the study findings and provided a more comprehensive assessment, however this was not undertaken due to logistics and resource limitations.

It has been previously suggested that due to possible sampling bias, the participants of the UKB study might not be representative of the general UK population, due to their better health-related parameters compared to the general UK population [30, 31]. We therefore might have underestimated the prevalence of iSS in the general population in the UK. The age of the UKB participants ranged from 40 to 69, and excluded other age groups in whom iSS may also occur. The reported prevalence of iSS may therefore have been different if younger (< 40 years old) or older (≥ 70 years) populations were included in the study. The exclusion of younger (< 40 years) and older (≥ 70 years) populations may have influenced the prevalence estimate as these groups could present with different iSS risk profiles. The true study prevalence may have been further skewed due to the selection bias as 35 scans were deemed of non-diagnostic quality due to motion artifacts which interfered with the interpretation of the imaging findings.

## Conclusion

We have estimated the prevalence of iSS from a large UK population sample. The findings are consistent with those from previous studies and suggest that iSS is more common than other well-recognized rare neurodegenerative diseases, identifying an important potential unmet healthcare need.

## Data Availability

The principal author takes full responsibility for the data, the analyses and interpretation, and the conduct of the research. The access to the data and its availability are regulated by the UKB.
